# Development and application of microsatellite markers in *Hippophae rhamnoides* subsp. *sinensis* Rousi (*Hippophae rhamnoides* L.) based on transcriptome sequencing

**DOI:** 10.3389/fgene.2024.1373028

**Published:** 2024-05-09

**Authors:** Qingqing Liu, Guisheng Ye, Yuhua Ma

**Affiliations:** ^1^ Agriculture and Animal Husbandry College, Qinghai University, Xining, Qinghai, China; ^2^ College of Animal Husbandry and Veterinary Science, Qinghai University, Xining, China

**Keywords:** *Hippophae rhamnoides* subsp. *sinensis* Rousi, SSR, primer development, genetic diversity evaluation, population genetic structure

## Abstract

*Hippophae rhamnoides* subsp. *sinensis* Rousi is a cold- and drought-tolerant pioneer species with significant economic and ecological value. Evaluating its genetic diversity and population structure is of great importance for guiding the development and utilization of resources. In this study, a total of 41,804 SSRs were generated by transcriptome sequencing of *Hippophae rhamnoides* subsp. *sinensis* Rousi. Among the different SSR motif types, mononucleotide repeats (26,972) were the most abundant, followed by trinucleotides, tetranucleotides, and pentanucleotides. 200 pairs of SSR primers were selected to detect polymorphisms, of which 15 pairs primers were selected as validated polymorphic SSRs used for genetic diversity and population structure analysis. A total of 63 alleles were identified with 15 pairs primers, with Nei’s genetic diversity index ranged from 0.27 to 0.83 (average: 0.54), and the expected heterozygosity ranged from 0.16 to 0.73 (average: 0.46). The polymorphism information content ranged from 0.23 to 0.81 (average: 0.48). Genetic structure analyses showed that the 10 populations could be broadly categorized into two groups. AMOVA denoted that genetic variations primarily originated from within the populations, with minimal differences observed between the groups, accounting for only 7% of the total genetic variation. This implies that mutation in *H. rhamnoides* subsp. *sinensis* Rousi mainly occurred within the populations. The results showed that the 10 populations of *H. rhamnoides* subsp. *sinensis* Rousi are rich in genetic diversity, with low levels of population differentiation and a high degree of gene exchange, which should be taken into consideration for the future work of germplasm resource preservation and seedling breeding.

## 1 Introduction

*Hippophae rhamnoides* subsp. *sinensis* Rousi (Chinese sea buckthorn) is a subspecies of *H. rhamnoides* L. with the largest distribution range and the longest cultivation history (Wang et al., 2014). It is widely distributed in Europe and Asia (Duan et al., 2022; Kaur et al., 2017; Mei et al., 2023; Ui Haq et al., 2021; Wang et al., 2021a). *Hippophae rhamnoides* subsp. *sinensis* has a well-developed root system with nodules, which can effectively improve the soil ecological environment and fertility (He et al., 2023). Additionally, it is a drought and cold-resistant species with a strong adaptive ability to soil, enabling it to survive and reproduce even in infertile gravel areas. Moreover, it can be utilized for water and soil conservation, windbreak, and sand fixation (Dong et al., 2020). The fruits of *H. rhamnoides* subsp. *sinensis* is rich in nutrients such as vitamins, linoleic acid, flavonoids, and various amino acids (Bal et al., 2011; Michel et al., 2011; Ghendov-Mosanu et al., 2022). Sea buckthorn oil, known as liquid gold, can effectively reduces cholesterol levels, protects the gastric mucous membrane (Trineeva et al., 2020; Pundir et al., 2021), prevents hypertension and hyperlipidemia, and promotes wound healing (Tuomasjukka et al., 2006). Therefore, the research and development of sea buckthorn can acquire excellent ecological, economic, and social value.

For a long time, due to environmental degradation and over-exploitation of natural resources, the stock of *H. rhamnoides* subsp. *sinensis* has declined dramatically. Thus, effective strategies are important to ensure the conservation and scientific utilization of *H. rhamnoides* subsp. *sinensis* (Dawson, 2011; Hu et al., 2021), and the diversity research is a prerequisite for the conservation of Chinese sea buckthorn resources. Recently, there have been a few studies on the genetic diversity of cultivated *H. rhamnoides* subsp. *sinensis* Rousi. From the morphological point of view, It found rich genetic diversity in *H. rhamnoides* subsp. *sinensis* across different regions, as evidenced by variations in morphological indexes such as leaf blade, fruit, tree height, crown width, and 100-fruit weight (Liu et al., 2023). Molecular analyses conducted by using ITS sequence analysis revealed genetic interpenetration between *H. rhamnoides* subsp. *sinensis* and other sea buckthorn subspecies, which contribute to the species diversity within *H. rhamnoides* subsp. *sinensis* Rousi (Jia et al., 2012). Additionally, Li Hongmei analyzed genetic variation in Chinese sea buckthorn through allelopathic enzymes, chromosomes, and morphology, the results showed that the degree of variation in *H. rhamnoides* subsp. *sinensis* differed between and within populations. Inter-population variation was mainly observed in traits such as 100-fruit weight, tepal shape, fruit shape, and seed thousand-grain weight, while intra-population variation among individuals mainly involved the number of thorns on 1-year-old shoots and the number of inflorescences bearing flowers (Li et al., 2003). It above research indicates the rich genetic diversity of Chinese sea buckthorn.

Transcriptome sequencing is widely used in the study of diversity research because of its wide dynamic range, sensitivity, precise, unbiased quantification of transcripts, and comprehensive coverage of all expressed sequences in a given tissue sample (Yang et al., 2018; Chakraborty et al., 2022; Liu et al., 2022). Simple-sequence repeats (SSRs), also known as microsatellites, are short tandem repeated motifs that may vary in the number of repeats at a given locus (Mahfooz et al., 2017). SSR markers have many advantages over other molecular markers, such as genetic co-dominance. They are multi-allelic, relatively abundant, widely dispersed across the genome, and easily and automatically scored (Gupta and Sharma, 2020; Yasar et al., 2020). SSR has become the best choice for DNA fingerprinting, gene mapping, variety identification, genetic diversity analysis and other applications. (Bhusan et al., 2016). Recently, SSRs have been widely employed in numerous studies on plant diversity, including research on species such as Camellia fascicularis (Bin et al., 2022), Beta vulgaris L. (Burns et al., 2010), Vanda stangeana Rchb. f. (Soneja et al., 2023), Oryza sativa L. (Li et al., 2023), or Punica granatum L. (Angelica et al., 2023). However, there is lack of the research on the genetic diversity of *H. rhamnoides* subsp. *sinensis* Rousi in Qinghai, and no published reports on the utilization of SSR markers, which makes it difficult to conduct in-depth studies on the genetic diversity of *H. rhamnoides* subsp. *sinensis* Rousi. The above studies show that Chinese sea buckthorn has rich genetic diversity. It is of great significance to study its genetic diversity. However, there is no systematic study on the development and application of ssr primers for Chinese sea buckthorn.

In this study, 15 pairs of SSR primers with clear bands, rich polymorphisms, and good repeatability were selected based on the transcriptome data of the *H. rhamnoides* subsp. *sinensis* (Ye et al., 2018). SSR molecular markers were analyzed on 150 samples from 10 districts in Qinghai Province. Our study aimed to develop polymorphic microsatellite markers and verify their polymorphism level for *H. rhamnoides* subsp. *sinensis* Rousi; and then evaluate the genetic diversity and structure among different populations using the polymorphic SSR markers from transcriptome sequencing. Our findings can provide valuable information for effective breeding and conservation strategies of *H. rhamnoides* subsp. *sinensis* Rousi germplasm resource.

## 2 Materials and methods

### 2.1 Experimental materials

**FIGURE 1:**
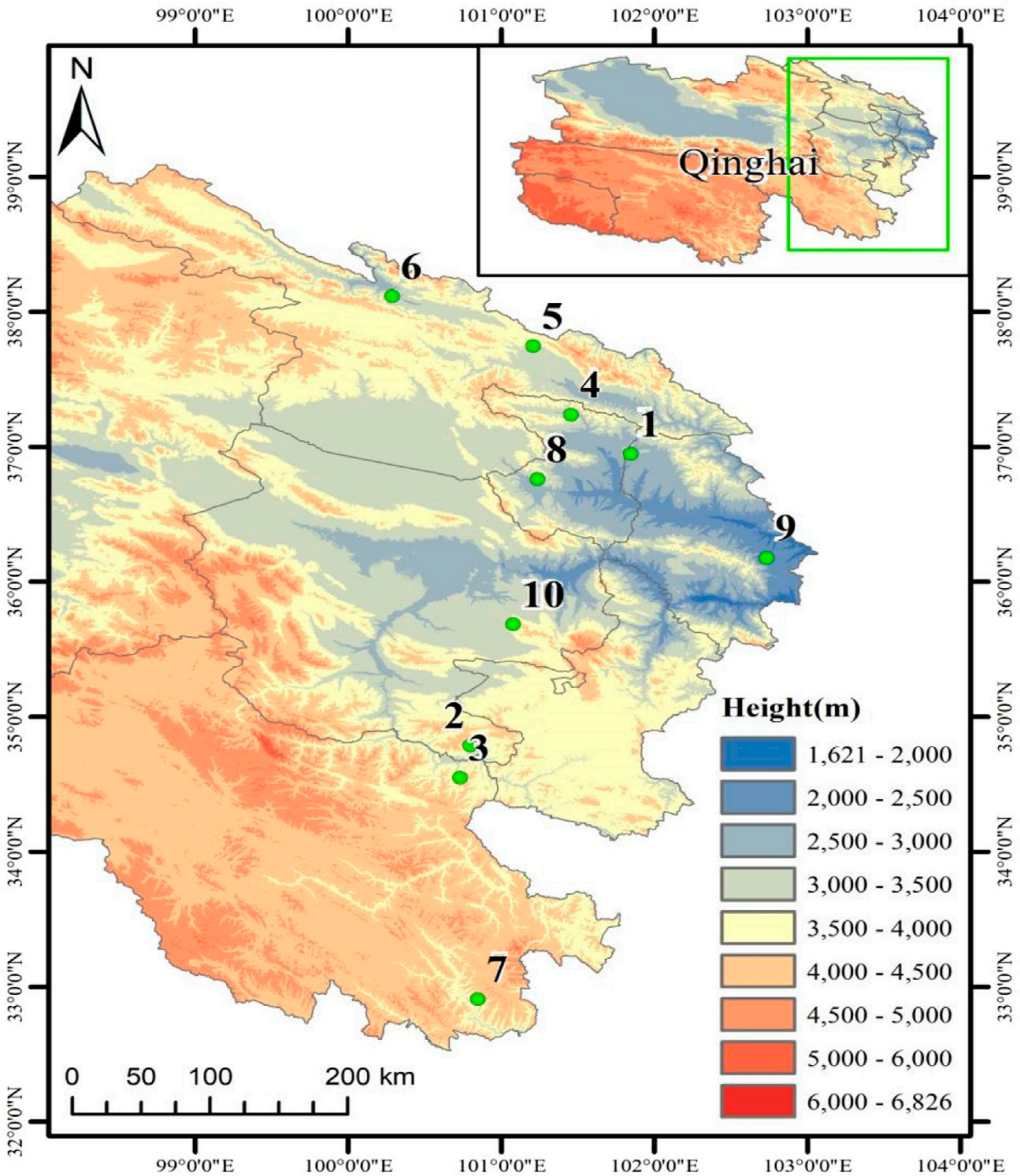
Sample collection map of *Hippophae rhamnoides* subsp. *sinensis*.

A total of 150 test samples from female plants of *H. rhamnoides* subsp. *Sinensis* Rousi without diseases and pests were collected from Menyuan, Maqin, Banma, Huzhu, Qilian, Tongde, Minhe, Huangyuan, Datong, and Guinan in Qinghai Province from August to September 2020 and 2021 respectively, with distances between individuals not less than 20 m. Fresh leaves were collected in separate ziplock bags and quickly dried with allochroic silica gel. The information of the test samples is shown in Table 1 and Figure 1.

**TABLE 1:** Sample information of *Hippophae rhamnoides* subsp. *sinensis*.

Populations	Location	Altitude(m)	Longitude (°E)	Latitude (°N)
1	Huzhu, Haidong City	2596	36.96	101.85
2	Tongde, Hainan City	3320	34.8	100.8
3	Maqin, Guoluo City	3350	34.56	100.73
4	Datong, Xining City	2468	37.25	101.46
5	Menyuan, Haibei City	2713	37.76	101.21
6	Qilian, Haibei City	3170	38.13	100.29
7	Banma, Guoluo City	3510	32.92	100.85
8	Huangyuan, Xining City	3010	36.77	101.24
9	Minhe, Haidong City	2080	36.19	102.74
10	Guinan, Hainan City	3540	35.7	101.08

### 2.2 The sourced of transcriptome data

Transcriptome data were derived from our previous experiments, and the specific method has been written in the previously published article (Ye et al., 2018).

### 2.3 DNA extraction

An adequate sample were taken for genomic DNA extraction according to the instructions of the modified CTAB Plant Genomic DNA Rapid Extraction Kit. The integrity and quality of the DNA were evaluated by electrophoresis on 0.8% agarose gels, and the concentration of the DNA was determined by NanoDrop One Spectrophotometer, and then all the DNA samples were diluted to 50 ng/μL and stored at −20°C for future use.

### 2.4 SSR primer-blast design and analyze

Primer3 was used to screen microsatellite loci and the primers were designed based on the identified SSR loci of the whole genome *H. rhamnoides* subsp. *Sinensis*. The SSR primers obtained from the systematic screening were further evaluated using oligo7 software to filter for hairpin structures, dimers, and mismatches. The main parameters of primer design were as follows: the length of primer was 18–28 bp, the temperature difference between the upstream and downstream primers was ≤5°C, and the annealing temperature of the primer pair was 50–65°C. The proportion of G and C bases ranged from 40%–70%; the fragment length of the amplified product was expected to be between 100 and 350 bp. According to the difference in Tm value and the size of the product, 200 pairs of primers were screened and synthesized. At the initial stage of the experiment, mixed samples of *H. rhamnoides* subsp. *sinensis* DNA were used for PCR with 200 pairs of primers. The effectively amplified bands of each pair of primers were examined.

### 2.5 SSR-PCR reaction system and program

The optimal PCR system developed by the project team was adopted: 0.8 U Taq enzyme, 2 mmol L^-1^ Buffer, 1.5 mmol·L^-1^dNTP, 0.5 umol·L^-1^ primer, 25 ng of template DNA, and 20 uL of ddH_2_O. The PCR amplification procedure was shown in Table 2, and the PCR reaction products were identified by 6% polypropylene gel electrophoresis and then the data were statistically analyzed.

**TABLE 2:** PCR amplification procedure.

Step	Condition		
1	Pre denaturation	95°C	5 min
2	Denaturation	95°C	1 min
	Anneal		30 s
	Extend	72°C	30 s
	Circulate	35cycle	
3	Extend	72°C	5 min
	Hold	4°C	∞

### 2.6 PCR amplification product detection of polymorphic primers and data analysis

Fifteen pairs of SSR primers of *H. rhamnoides* subsp. *sinensis* were selected for the marker analysis of 150 samples from 10 natural populations. Popgene32 statistics were used to calculate the number of observed alleles (Na), the number of effective alleles (Ne), Shannon’s information index (I), Observed homogeneity (Obs_Hom), Observed heterozygosity (Obs_Het), Expected homogeneity (Exp_Hom), Expected heterozygosity (Exp_Het), Diversity index Nei, genetic similarity and genetic distance. Genetic diversity indices were obtained by analyzing the genetic diversity and genetic differentiation of 15 pairs of SSR primers and 150 Chinese sea buckthorn samples by the Excel 2010 add-in GenAlEx 6.51b2 (Peakall, 2012). The analysis of molecular variance (AMOVA) between and within different populations of the *H. rhamnoides* subsp. *sinensis* was performed by GenAlEx 6.51b2. The polymorphism information content (PIC) of SSR loci of Chinese sea buckthorn was analyzed with Power Marker software, while Nei’s genetic distances were calculated among different populations. A clustering tree was constructed based on Nei’s genetic distances in MEGA X. STRUCTURE 2.2.3 software and used to analyze the genetic structure of the 10 populations of Chinese sea buckthorn. To obtain the best k value, CLUMPP software was used to merge the data, DISTRUCT software was used to obtain the structural map under the best k value, and AI software was used to obtain the final result map.

## 3 Results

### 3.1 Transcriptome assembly and sequence annotation

The raw reads produced in this study have been deposited in the Short Read Archive of the National Center for Biotechnology Information (NCBI) with accession numbers SRR7003894, SRR7003893, SRR7003896, SRR7003895, SRR7003892, and PRJNA449450. The articles published in the early stage of transcriptome assembly and gene annotation have been analyzed in detail (Ye et al., 2018). Which include GO and KEGG pathway enrichment analysis.

**FIGURE 2:**
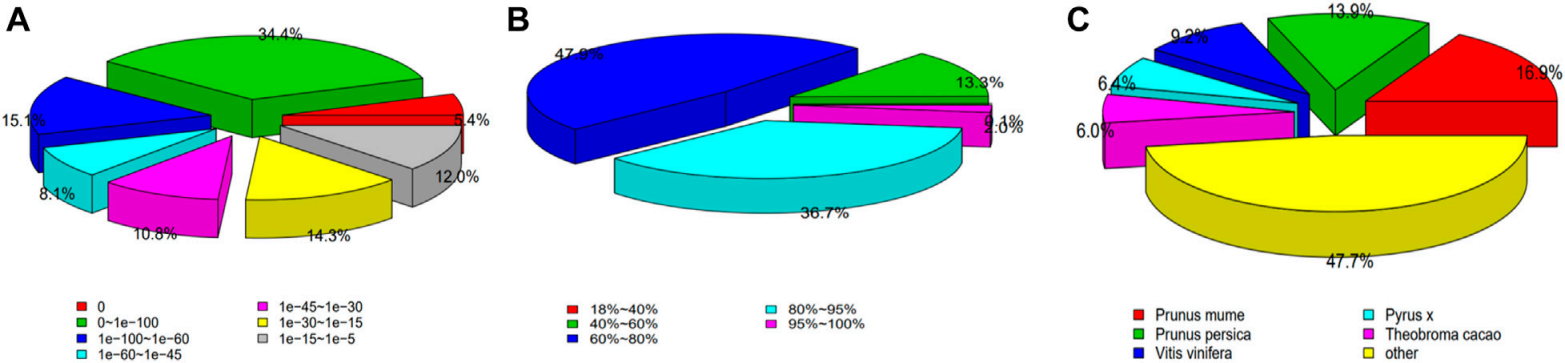
Nr Annotation of the *Hippophae rhamnoides* subsp. *sinensis*. **(A)** E-value distribution. **(B)** similarity distribution. **(C)** species classification.

The Nr annotation results showed that E-value ≤0. Similarity distribution showed that more than 85% of sequences with more than 60% similarity, which indicates a high degree of confidence in these results. Similarity classifications showed that *H. rhamnoides* subsp. *sinensis* had the highest similarity with Prunus mume (16.9%) and the second highest similarity with Prunus persica (13.9%), indicating that *H. rhamnoides* subsp. *sinensis* and Prunus mume are most closely related (Figure 2).

### 3.2 Development of the SSRs based on transcriptome sequencing

**FIGURE 3:**
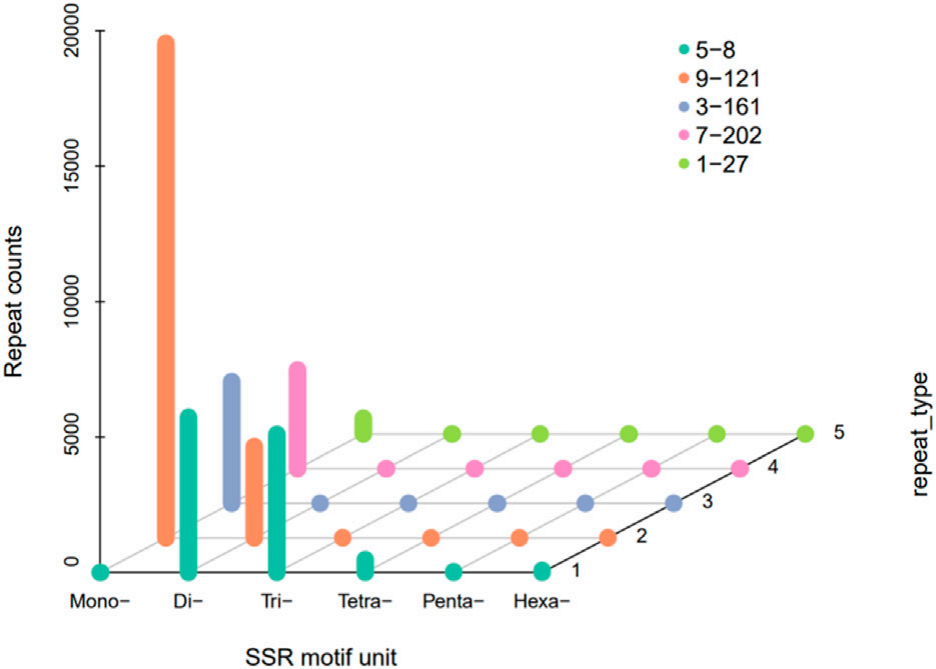
The number distribution of SSR motif unit in *Hippophae rhamnoides* subsp. *sinensis*. transcriptome.

141,380,000 bp in Genome discovered by MISA and 32,709 sequences contained a total of 41,804 SSR sites, of which 7,148 sequences contained 1 SSR site. The genome of *H. rhamnoides* subsp. *sinensis*. Has a rich variety of SSR nucleotide repeating motifs, and the repeating motifs have base variations ranging from 1 to 6. The mononucleotide SSR motifs were the most abundant type of repeats (26,976), accounting for 64.53% of the total SSRs, followed by the dinucleotide, trinucleotide, tetranucleotide, pentanucleotide, and hexanucleotide SSR motifs, and the penta-type was the least abundant (Table 3; Figure 3).

**TABLE 3:** SSR loci information in *Hippophae rhamnoides* subsp. *sinensis*.genome.

Category	Number
Total size of examined sequences (bp)	141,380,000
Total number of identified SSRs	41,804
Number of SSR-containing sequences	32,709
Number of sequences containing more than 1 SSR	7,148
Number of SSRs present in compound formation	2,536
The number of mono-nucleotide	26,976
The number of di-nucleotide	9,102
The number of tri-nucleotide	5,111
The number of tetra-nucleotide	480
The number of penta-nucleotide	39
The number of hexa-nucleotide	96

**FIGURE 4:**
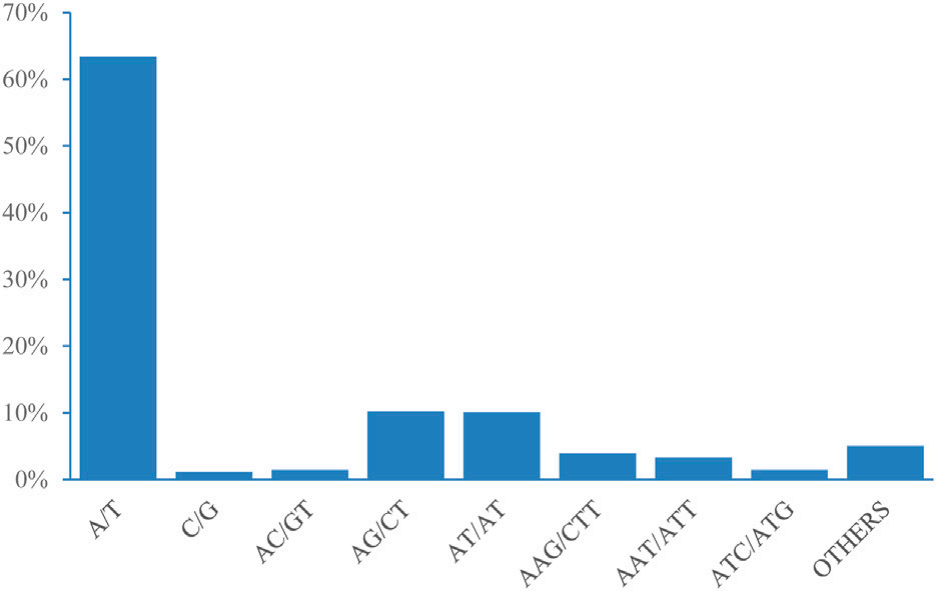
Frequency distribution of genome-SSRs based on motif numbers.

According to the length of the genomic SSRs based on the number of repeat units (Table 4), the most abundant type of repeats was ten, accounting for 13,235 (31.66%), followed by eleven, six, five, and seven repeat units. Only 1,332 units presented more than 20 repeats (3.19%). The five, six, seven, ten, and eleven repeat units accounted for 65.52% of the total repeat units, which could explain the predominant diversity of SSR repeat unit types. The most abundant type of repeat motif was A/T, accounting for 26,502 of the repeats (63.40%), followed by AG/CT, AT/AT, and AAG/CTT, the other SSR motifs types (2,108) accounted for 5.04% of the repeats (Figure 4).

**TABLE 4:** Frequency of SSR motifs of genome-SSRs in *Hippophae rhamnoides* subsp. *sinensis*.

Motif length	Number of repeats															
	5	6	7	8	9	10	11	12	13	14	15	16	17	18	19	≥20
mono-						11,916	4,092	2,251	1,534	1,138	1,018	801	941	955	1,002	1,328
di-		2,554	1,549	1,628	1,734	1,310	317	9	1							
tri-	2,580	1,473	1,005	43	2	4	1	1				1				1
tetra-	426	49	1	3			1									
penta-	19	5	2	6	2			1	2							2
hexa-	58	13	5	6	2	5	3		1	2						1

### 3.3 Polymorphism primer

From the *H. rhamnoides* subsp. *sinensis* SSR primer sequences designed by Primer3 software, 200 pairs of qualified primers were randomly selected and sent to Shanghai Sangong Bioengineering Co., Ltd. for synthesis. Based on the optimized SSR system, the synthesized primers were used to amplify the *H. rhamnoides* subsp. *sinensis* samples, finally, 90 pairs of primers were able to amplify clear bands, which were then screened by polypropylene gel electrophoresis. A total of 15 pairs of polymorphic primers were selected (Table 5).

**TABLE 5:** Sequence of SSR primers.

ID	Code	Forward Primer (5′-3′)	Reverse Primer (5′-3′)	Repeat motif	Amplicon size	Tm (°C)
Cluster-17196.24404	189	ACT​ACC​CAT​TTG​CAG​AGC​CC	TGC​TAC​TCC​ATG​TCA​CAG​CT	(T)10	228	55.5
Cluster-17196.16325	81	GGC​TCT​GGA​GGC​CAA​TGT​TT	AGG​AAG​TTG​TGC​CGT​ACC​TG	(TTC)5	225	54
Cluster-17196.58805	18	TCC​TCC​ACA​ACC​ATT​TCG​CT	GAG​ACG​GCT​CTT​CGG​ATC​TG	(TTC)5	170	55.8
Cluster-17196.96526	141	TGA​TCT​CGT​CCT​GTT​CAT​TCA​CT	CGC​TAG​CTC​GTA​ATT​GTA​GTT​GC	(T)10	280	53.5
Cluster-17196.55903	105	TGT​CTC​TGT​CTA​CTG​CCC​TT	ACT​ATG​AGA​TCT​AGC​CGC​CT	(TA)7	146	49
Cluster-17196.24714	157	TCA​GGA​CCG​ACT​TTG​CAT​GG	TGG​GGT​TCT​CCT​AGA​ATG​GT	(T)10	133	53.6
Cluster-17196.38391	68	CGT​GGG​CCA​ATG​ACA​CTT​TG	TAG​GGC​CTT​TTG​TGC​CTC​TC	(T)11	177	55
Cluster-17196.48788	109	GGG​CAG​AAA​TGG​TGA​AGG​CT	ACC​CAC​CAG​CAA​TTT​CAT​GC	(AG)8	209	55.8
Cluster-17196.78862	11	TGC​AAG​AAA​CCA​GGT​GAG​CT	ACG​TCC​CCG​GAA​TCA​CTT​TC	(A)11	169	54
Cluster-16179.3	133	TGT​CCA​CCA​AAC​TCT​CAC​CG	GCG​AGA​TCC​CCG​AGT​TAC​AG	(GAA)6	135	53.5
Cluster-17196.39117	156	CAC​CAC​CGC​TTC​TCT​TCC​TC	AAA​CCG​ACT​TCC​CAC​TTC​CC	(TC)6	279	54
Cluster-17196.14403	146	ACT​GGG​GTG​AAA​CTG​ACG​AC	AAG​GGG​CAT​GGC​AGT​ACT​TC	(TA)6	128	55.5
Cluster-17196.51109	180	TAC​TCC​CAA​GCT​CCT​CCT​CC	GGC​TGC​GTT​TGA​TAT​TTG​CG	(TC)8	243	52.6
Cluster-17196.48626	121	ATC​CAA​GTC​CTC​AGC​CAA​GC	GGC​CGC​CTT​TTA​CTA​ACC​CT	(TCT)5	198	55.5
Cluster-17196.37024	185	GTA​CAA​ACG​GCG​ATG​ATG​GC	ATG​CAC​TGG​AAG​TCA​GAG​GC	(CAA)5	198	55

### 3.4 Primer polymorphism analysis

200 pairs of primers were used to screen polymorphic primers, of which 15 pairs of primers could produce clear bands and rich polymorphisms, and then were used to detect the genetic diversity of 150 *H. rhamnoides* subsp. *sinensis* Rousi samples from 10 populations (Table 6). 63 alleles were identified, ranging from 2 to 10 (NA), among them, primer 156 amplified the most allelic sites (10), 189, 141, 68, and 11 amplified the least allelic sites (2). The Ne ranged from 1.37 to 5.99. The I varied from 0.44 to 1.95 (mean: 0.98). The Exp_Hom varied from 0.16 to 0.73 (mean: 0.46). The Obs_Hom varied from 0.01 to 0.76, (mean: 0.34). The Exp_Het varied from 0.27 to 0.84 (mean: 0.54). The Obs_Het varied from 0.24 to 0.99 (mean: 0.66). This indicates that the test material has a high degree of heterozygosity. The average number of effective alleles was 2.51, and the diversity index Nei ranged from 0.27 to 0.83 (mean:0.54). The PIC ranged from 0.23 to 0.81 (mean: 0.48), among which there were 5 pairs of primers with PIC>0.5 and 9 pairs of primers with PIC values between 0.25 and 0.5. When the PIC is greater than 0.25, it indicates that the locus is medium polymorphic. It can be seen that 93.3% of the primers had medium to high polymorphism, indicating that the SSR primers selected in this study were highly polymorphic.

**TABLE 6:** Statistical analysis of polymorphic parameters of the 15 SSR primer.

Code	Na	Ne	I	Exp_Hom	Obs_Hom	Exp_Het	Obs_Het	Nei	Pic
189	2	1.97	0.68	0.51	0.15	0.49	0.85	0.49	0.37
81	7	2.72	1.23	0.37	0.23	0.63	0.77	0.63	0.58
18	3	2.39	0.96	0.42	0.07	0.58	0.93	0.58	0.5
141	2	1.9	0.67	0.53	0.46	0.47	0.54	0.47	0.36
105	3	1.76	0.76	0.57	0.45	0.43	0.55	0.43	0.39
157	3	2.19	0.91	0.46	0.22	0.54	0.78	0.54	0.48
68	2	1.61	0.57	0.62	0.53	0.38	0.47	0.38	0.31
109	7	3.59	1.48	0.28	0.17	0.72	0.83	0.72	0.68
11	2	1.37	0.44	0.73	0.76	0.27	0.24	0.27	0.23
133	3	2.08	0.78	0.48	0.01	0.52	0.99	0.52	0.4
156	10	5.99	1.95	0.16	0.2	0.84	0.8	0.83	0.81
146	4	2.89	1.19	0.34	0.17	0.66	0.83	0.65	0.59
180	5	2.19	1.06	0.45	0.65	0.55	0.35	0.54	0.5
121	6	3.7	1.43	0.27	0.31	0.73	0.69	0.73	0.68
185	4	1.38	0.59	0.72	0.75	0.28	0.25	0.27	0.26

Na, Number of alleles; Ne, number of effective alleles; I, shannon information index; Exp_Hom, expected homozygosity; Obs_Hom, observed homozygosity; Exp_Het, expected heterozygosity; Obs_Het, observed heterozygosity; Nei, diversity Sex index.

### 3.5 Genetic diversity within populations

The genetic diversity of *H. rhamnoides* subsp. *sinensis* Rousi from different populations were analyzed, and all parameters of the 15 SSR loci were calculated (Table 7). The Na ranged from 2.67 to 3.40, with an average of 3.13; Ne ranged from 1.91 to 2.42, with an average of 2.25; Na and Ne indicated that there were allelic differences among Chinese sea buckthorn populations, but the genetic differences were not significant. The highest I was 0.90, the lowest was 0.66, and the average was 0.83; the He ranged from 0.40 to 0.51, with an average of 0.49; the Ho ranged from 0.58 to 0.73, with an average of 0.66; the uHe ranged from 0.42 to 0.53, with an average of 0.50; the HO was greater than the He has shown the majority of SSR loci had null alleles. Population 7 had the highest genetic diversity in all the populations (Na = 3.40, I = 0.90, He = 0.51). In the selection and breeding of *H. rhamnoides* subsp. *sinensis* Rousi, priority can be given to plants in Population 7.

**TABLE 7:** Genetic diversity analysis of *Hippophae rhamnoides*.

Pop	N	Na	Ne	I	Ho	He	uHe	F
1	15	3.13	2.19	0.80	0.60	0.46	0.48	−0.24
2	15	2.67	1.91	0.66	0.58	0.40	0.42	−0.41
3	15	3.13	2.21	0.85	0.68	0.49	0.51	−0.37
4	15	3.20	2.27	0.83	0.65	0.49	0.51	−0.32
5	15	3.27	2.42	0.89	0.71	0.51	0.53	−0.36
6	15	3.07	2.34	0.86	0.68	0.51	0.53	−0.29
7	15	3.40	2.39	0.90	0.61	0.51	0.53	−0.16
8	15	3.27	2.34	0.88	0.68	0.50	0.52	−0.37
9	15	3.20	2.22	0.81	0.64	0.47	0.49	−0.30
10	15	3.00	2.20	0.84	0.73	0.51	0.53	−0.42

Pop, sample size; Na, Number of alleles; Ne, number of effective alleles; I, Shannon’s information index; Ho, observed heterozygosity; He, expected heterozygosity; uHe, unbiased expected heterozygosity; F, fixation index. The populations represented by each cluster number are 1: Huzhu, 2: Tongde, 3: Maqin, 4: Datong, 5: Menyuan, 6: Qilian, 7: Banma, 8: Huangyuan, 9: Minhe, 10: Guinan.

### 3.6 Analysis of population genetic differentiation

Inbreeding coefficients (Fis) ranged from −0.91 to 0.24 (Table 8) with an average value less than 0, indicating that there were excessive heterozygotes in the *H. rhamnoides* subsp. *sinensis* Rousi populations. The coefficient of inbreeding (Fit) ranged from −0.90 to 0.36, the coefficient of genetic differentiation (Fst) ranged from 0.01 to 0.21, and the minimum value of gene flow (Nm) was 0.93 and the maximum value was 45.35, which indicated that gene flow was at a relatively high level among Chinese sea buckthorn populations, and that a large number of genes were exchanged among populations.

**TABLE 8:** Correlation coefficient of population genetic differentiation.

Locus	Fis	Fit	Fst	Nm
189	−0.84	−0.74	0.06	4.28
81	−0.54	−0.21	0.21	0.93
18	−0.71	−0.61	0.06	3.93
141	−0.2	−0.14	0.05	4.74
105	−0.39	−0.27	0.08	2.71
157	−0.72	−0.44	0.16	1.28
68	−0.31	−0.23	0.06	4.16
109	−0.23	−0.16	0.06	3.70
11	0.00	0.11	0.10	2.16
133	−0.91	−0.90	0.01	45.35
156	−0.04	0.04	0.08	2.94
146	−0.36	−0.26	0.07	3.25
180	0.24	0.36	0.16	1.28
121	−0.11	0.06	0.15	1.37
185	0.05	0.10	0.06	4.21

Genetic distance and genetic concordance are two important indicators used to determine the kinship between populations. A genetic similarity of zero suggests complete dissimilarity and unrelatedness between populations, while a genetic similarity of 1 indicates complete identity. The magnitude of the genetic distance directly reflects the degree of relatedness. In this study, the genetic consistency ranged from 0.80 to 0.96, and the genetic distance ranged from 0.04 to 0.22 (Table 9), indicating that the 10 populations were relatively close to each other. Among them, the genetic similarity between the population 9 and the population 5 was the highest (0.96), and the genetic distance was the smallest (0.04). It suggests that the population 9 and the population 5 are the least genetically differentiated and most closely related.

**TABLE 9:** Genetic consistency (upper right triangle) and genetic distances (lower left triangle) of *Hippophae rhamnoides* subsp. *sinensis*.

Pop	1	2	3	4	5	6	7	8	9	10
1	****	0.90	0.91	0.93	0.93	0.90	0.87	0.89	0.90	0.86
2	0.10	****	0.90	0.85	0.86	0.83	0.80	0.88	0.85	0.84
3	0.09	0.11	****	0.92	0.91	0.87	0.85	0.89	0.89	0.86
4	0.07	0.16	0.08	****	0.92	0.90	0.87	0.88	0.87	0.82
5	0.08	0.15	0.10	0.09	****	0.94	0.89	0.94	0.96	0.94
6	0.10	0.18	0.13	0.10	0.06	****	0.91	0.88	0.92	0.89
7	0.14	0.22	0.17	0.14	0.11	0.09	****	0.86	0.88	0.88
8	0.12	0.13	0.11	0.13	0.06	0.13	0.15	****	0.95	0.93
9	0.10	0.16	0.12	0.13	0.04	0.08	0.13	0.05	****	0.94
10	0.16	0.17	0.15	0.20	0.06	0.11	0.13	0.07	0.06	****

The populations represented by each cluster number are: 1: Huzhu, 2: Tongde, 3: Maqin, 4: Datong, 5: Menyuan, 6: Qilian, 7: Bama, 8: Huangyuan, 9: Minhe, 10: Guinan.

### 3.7 Analysis of molecular variance (AMOVA) of the settlements

The Analysis of molecular variance results are shown in Table 10. AMOVA revealed that 7% of the total variation was due to differences among populations, with the remaining 97% due to differences within populations, the genetic variation rate within the populations was much larger than that between populations, which indicated that the genetic variations of *H. rhamnoides* subsp. *sinensis* in Qinghai Province mainly occurred in the individuals of the populations. Intra-individual variation refers to genetic differences caused by heterozygous alleles, the size of which correlates with the number of individual heterozygous, the genetic diversity of individual suggesting that there is a high degree of genetic differentiation within individual, and that individual genetic variation is the main source of *H. rhamnoides* subsp. *sinensis* Rousi variation.

**TABLE 10:** Molecular analysis of variance within and between populations.

	DF	SS	MS	VC	Total variation
Among Pops	9	115.67	12.85	0.34	0.07
Among Indiv	140	356.33	2.55	0	0
Within Indiv	150	739	4.93	4.93	0.93
Total	299	1,211.00	20.33	5.27	1

DF, degrees of freedom; SS, sum of squares; MS, mean square error; VC, variance component; Total variation, percentage of total variation.

### 3.8 Population principal coordinate analysis (PCoA)

**FIGURE 5:**
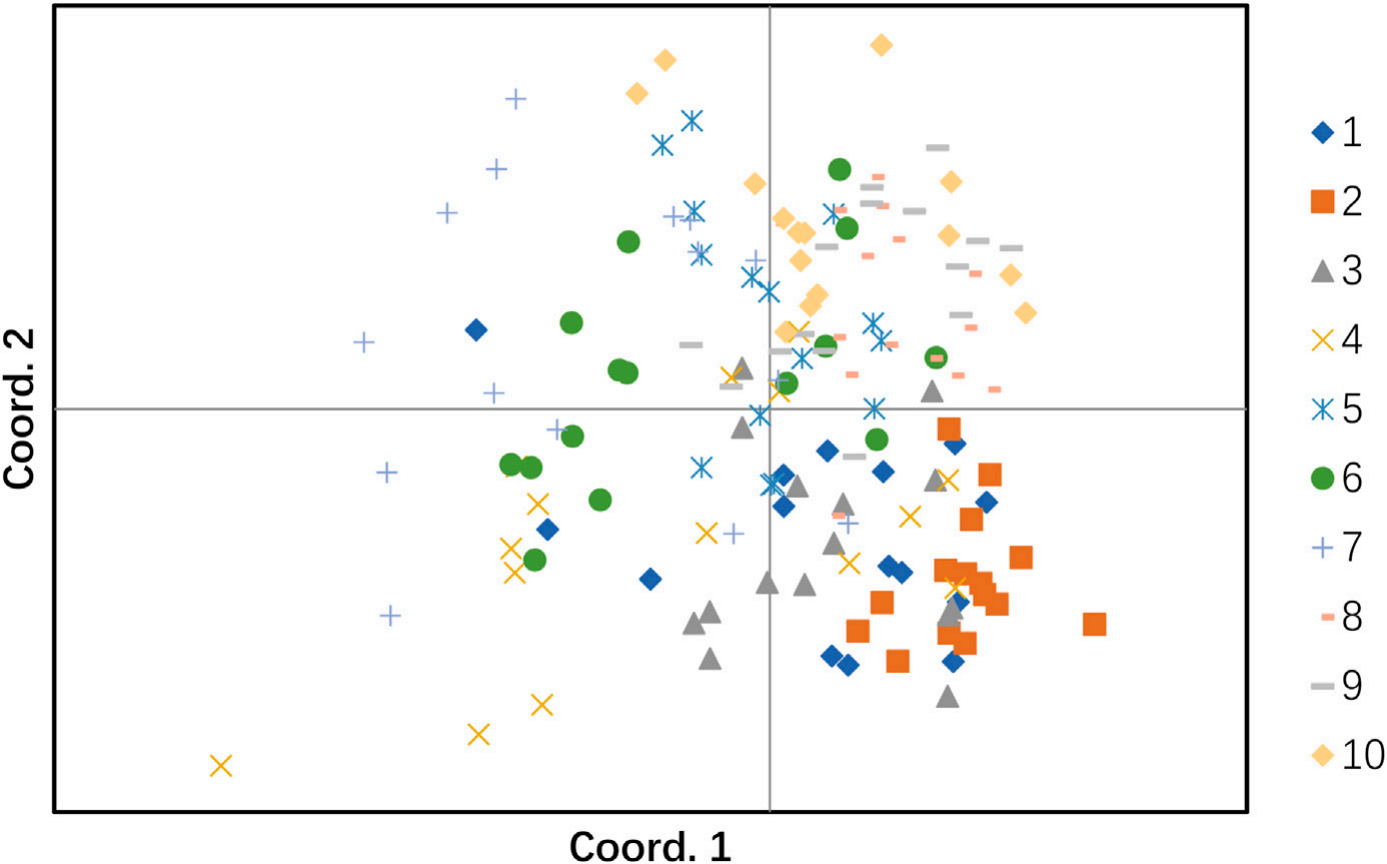
The principal coordinate analysis (PCoA). The cluster numbers represent the following: 1: Huzhu, 2: Tongde, 3: Maqin, 4: Datong, 5: Menyuan, 6: Qilian, 7: Banma, 8: Huangyuan, 9: Minhe, 10: Guinan.

To further study the genetic similarity and evolution of different populations of *H. rhamnoides* subsp. *sinensis* and to visualize the results clearly and concisely, the GenAlEx6.51b2 software was used to analyze PCoA of 10 populations based on Nei’s genetic distance. The eigenvalues of the three principal coordinates contributing to the total variation accounted for 31.13%, of which the first principal coordinate accounted for 12.27% of the total variation, the second principal coordinate accounted for 10.64% of the total variation, and the third principal coordinate explained 8.23% of the total variation (Table 11). The PCA analysis revealed two groups of samples, of which one group included populations 1, 2, 3, and 4. Because they are more concentrated in the lower half of the plot. The other group included populations 5, 6, 7, 8, 9 and 10, which are more concentrated in the upper half of the plot (Figure 5).

**TABLE 11:** Percentage change explained by the first three axes.

Axis	1	2	3
%	12.27	10.64	8.23
Cum %	12.27	22.91	31.13

### 3.9 Cluster analysis

**FIGURE 6:**
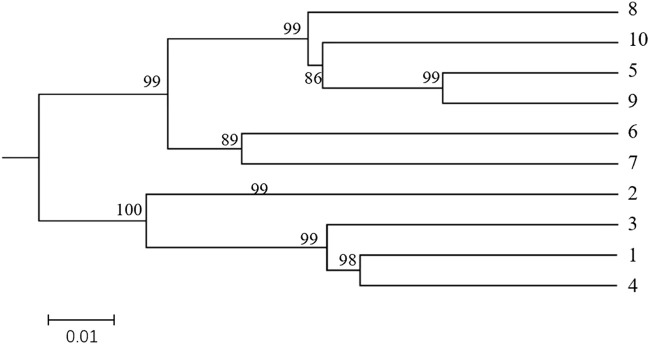
Dendrogram of the *Hippophae rhamnoides* subsp. *sinensis* Rousi populations constructed using UPGMA methods UPGMA methods based on matrices of pairwise Nei′s genetic distances. Note:The clusters represented by the numbers are: 1: Huzhu, 2: Tongde, 3: Maqin, 4: Datong, 5: Menyuan, 6: Qilian, 7: Banma, 8: Huangyuan, 9: Minhe, 10: Guinan.

The UPGMA clustering diagram were constructed using MEGAX software based on Nei’s genetic distance among different populations of *H. rhamnoides* subsp. *sinensis* Rousi, as shown in Figure 6. The bootstrap value of each arm are greater than 80%, indicating that the results are credible. The 10 populations were divided into two groups: populations 1, 2, 3, and 4 formed the first group, while populations 5, 6, 7, 8, 9, and 10 constituted the second group, consistent with the PCA results.

### 3.10 Population genetic structure analysis

**FIGURE 7:**
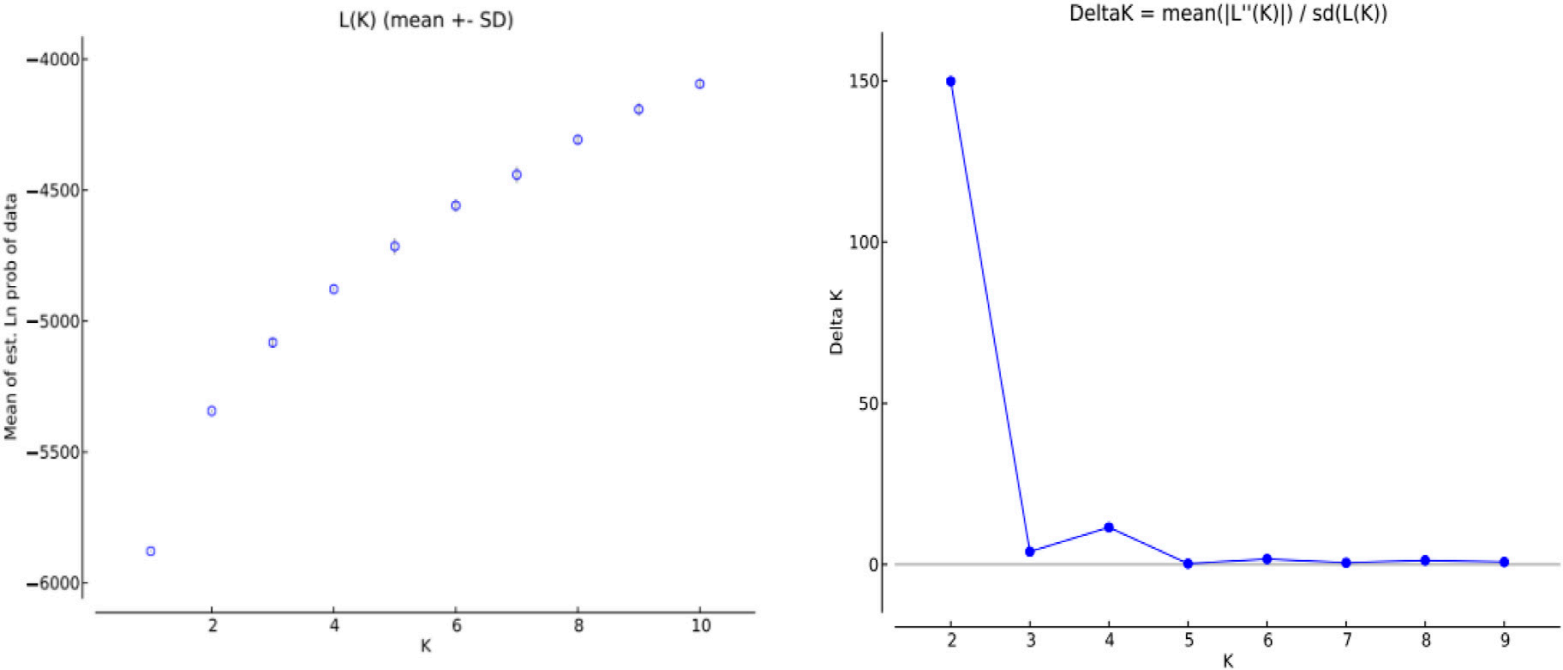
Variation law of ΔK value with K value.

**FIGURE 8:**
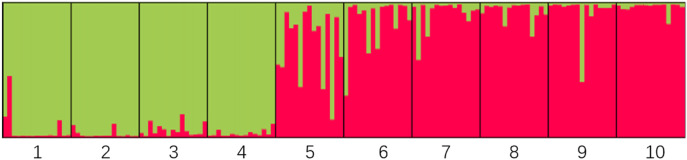
Structure of optimal K value. Note: The residence groups represented by each cluster number are: 1: Huzhu, 2: Tongde, 3: Maqin, 4: Datong, 5: Menyuan, 6: Qilian, 7: Banma, 8: Huangyuan, 9: Minhe, 10: Guinan.

The genetic structure of 10 *H. rhamnoides* subsp. *sinensis* Rousi populations were analyzed using STRUCTURE 2.2.3 software, setting the range of the group number K values as 1 to 10, repeating the operation 10 times for each K value, and plotting the relationship between K values and ΔK. submitted to the website http://taylor0.biology.ucla.edu/structure Harvester/for analysis. The structure of *H. rhamnoides* subsp. *sinensis* Rousi germplasm resource genotypes was analyzed based on the likelihood of data [LnP(D)]. The germplasm resources were integrated into groups (K) to assess the variant frequency of each group, and the individual germplasms were reintegrated into groups based on the estimated frequencies. The number of subgroups varies with the LnP(D) values. The curve occurs between K = 1 and K = 2, and all the values are at their maximum. (Table 12, Figure 7). Therefore, the 150 accessions could be divided into two subgroups. The outputs of K = 2 were visualized through CLUMPP and DISTRUCT. Then, we visualized that all individuals are clustered into two groups, including the wild group (green) and the cultivated group (red) (Figure 8). The first group consisted of clusters from Huzhu, Tongde, Maqin, and Datong, and the second group consisted of clusters from Mengyuan, Qilian, Banma, Huangyuan, Minhe and Guinan. This is consistent with the results of MEGAX software clustering.

**TABLE 12:** Parameter values under different K values.

K	Reps	Mean LnP(K)	Stdev LnP(K)	Ln'(K)	|Ln''(K)|	Delta K
1	10	−5878.89	0.78	—	—	—
2	10	−5343.37	1.83	535.52	274.24	149.86
3	10	−5082.09	14.47	261.28	57.74	3.99
4	10	−4878.55	3.46	203.54	39.93	11.53
5	10	−4714.94	28.93	163.61	7.93	0.27
6	10	−4559.26	22.03	155.68	37.76	1.71
7	10	−4441.34	28.77	117.92	16.00	0.56
8	10	−4307.42	14.15	133.92	18.16	1.28
9	10	−4191.66	23.73	115.76	18.56	0.78
10	10	−4094.46	16.94	97.2	—	—

The cluster numbers represent the neighborhoods: 1: Huzhu, 2: Tongde, 3: Maqin, 4: Datong, 5: Menyuan, 6: Qilian, 7: Banma, 8: Huangyuan, 9: Minhe, 10: Guinan.

## 4 Discussion

*Hippophae rhamnoides* subsp. *sinensis* is a typical plant with significant ecological and economic value. With the rapid advancement of high-throughput sequencing technology, scholars have analyzed the transcriptome of *H. rhamnoides* subsp. *sinensis* Rousi. Zhou et al. used RNA-Seq high-throughput sequencing to obtain transcriptome data from the leaves of male and female *H. rhamnoides* subsp. *sinensis* Rousi plants, identifying 33,248 microsatellite markers, which provided the basic data for gene mining and molecular labeling of Chinese sea buckthorn (Zhou et al., 2019). In this study, the transcriptunigene sequence count reached 175,425, with a total of 41,804 SSR sites identified. The SSR repeat types were abundant, and the content of short repeat units was high in the Chinese sea buckthorn transcriptom, which was consistent with the results of Wang et al. (Wang et al., 2021b). This study provides new high-throughput data for transcriptomics, which provides valuable information for germplasm conservation and breeding of *H. rhamnoides* subsp. *sinensis* Rousi. The Nr results showed that Chinese sea buckthorn had the highest sequence similarity with Prunus mume, but the SSR primers of Prunus mume can not applied to *H. rhamnoides* subsp. *sinensis* Rousi (Xue, 2021), because the SSR sequences obtained in this research had more sites and appeared more frequently. It indicates that SSRs are variable in different plants, and this situation is mainly caused by different development methods, search criteria, and the abundance of DNA sequence resources (Wei et al., 2011; Xie et al., 2023).

Currently, the development of SSR markers using transcriptome sequences has been further extended and has been widely used in population genetics, the construction of genetic linkage maps, and analysis of genetic relationships between germplasm and marker-assisted selection breeding (Gong, et al., 2016; Tulsani, et al., 2020; Tian, et al., 2021; Duan, et al., 2022a), RNA-Seq has become an effective method for analyzing transcriptome data. SSR markers have also been developed and applied by Chinese sea buckthorn RNA-seq by previous authors. Li et al. used 17 polymorphic RNA-Seq SSR primers to study the genetic variation and population genetic structure of 80 *H. rhamnoides* subsp. *sinensis* Rousi from Datong and Dongsheng, found that the genetic diversity of Chinese sea buckthorn was consistent with their origin and genetic background (Li, et al., 2019). Wang et al. found that the genetic diversity of *H. rhamnoides* subsp. *sinensis* Rousi was higher than that of Yunnan sea buckthorn by using RNA-Seq SSR primers (Wang, et al., 2021b). However, due to the late start of the application of SSR based on transcriptome sequencing in Chinese sea buckthorn, it is difficult to develop SSR primers.

Chinese sea buckthorn is wind-pollinated and dioecious, combined with the wide distribution area, the differences in ecological factors such as climate, soil and altitude in the distribution area, as well as the evolution of the population, forming a rich variation type of plants in the genus (Ma et al., 2014). Qinghai is the birthplace of seabuckthorn, it is of great significance to study the genetic variation of Chinese seabuckthorn. The ssr primers developed in this study can provide the basis for the genetic diversity of Chinese sea buckthorn in Qinghai. 15 pairs of SSR primers designed were able to amplify clear and polymorphic bands. The reason why the remaining primers could not be amplified may be: (1) There are many intronic fragments in the genomic DNA, and one or both ends of the primers designed for the SSR site are located at the intronic or exonic shear point, so the primers could not find the binding site in the genome (Gao, 2013). (2) In the initial screening of primers, PCR was carried out by uniformly annealing temperature of 55°C, which may lead to the failure of those primers with lower annealing temperature.

In this study, the genetic diversity of 10 *H. rhamnoides* subsp. *sinensis* Rousi populations were analyzed and the results showed that the number of alleles detected in SSRs was 31.34, with an average of 3.13 alleles per locus. At the population level, the Shannon information index of Chinese sea buckthorn ranged from 0.66 to 0.90, indicating high genetic diversity. Wu Q (Wu et al., 2007) analyzed the phenotypic traits of *H. rhamnoides* subsp. *sinensis* Rousi in Wutai Mountain, Shanxi Province, showed that Chinese sea buckthorn at the same altitude and habitat had a wide range of morphological traits, which were rich in genetic diversity. Tian (Tian et al., 2004) analyzed its genetic diversity by ISSR and found that *H. rhamnoides* subsp. *sinensis* Rousi was very rich in genetic diversity. The above results consistent with ourresearch results and indicate the genetic diversity was very rich in Chinese sea buckthorn form different population. It can be shown that the developed primers have a high level of polymorphism. The test material has rich genetic diversity, which can provide certain theoretical references for the conservation, development and utilization of the excellent germplasm of *H. rhamnoides* subsp. *sinensis* Rousi.

Both the clustering tree and STRUCTURE 2.2.3 software classified the 10 populations of *H. rhamnoides* subsp. *sinensis* Rousi into two categories. The first group consisting of wild populations from Huzhu, Tongde, Maqin, and Datong. The second group consisting of populations from Menyuan, Qilian, Banma, Huangyuan, Minhe, and Guinan. The genetic diversity of *H. rhamnoides* subsp. *sinensis* Rousi on both sides of the Qilian Mountains found that the geographical isolation of the Qilian Mountains led to restricted gene exchange among *H. rhamnoides* subsp. *sinensis* Rousi populations, thus forming obvious genetic differentiation (Zhang et al., 2006). The genetic variation and structure of wild populations of *H. rhamnoides* subsp. *sinensis* Rousi using cpSSR and RAPD showed varying degrees of genetic differentiation among populations from different regions (Zhao, 2007). DNA molecular markers found that *H. rhamnoides* subsp. *sinensis* Rousi has rich genetic diversity due to the influence of altitude and geographical distance, indicating that geographical isolation and gene exchange were the main factors affecting its genetic diversity. (Sun et al., 2013). However, in this study, the genetic diversity of *H. rhamnoides* subsp. *sinensis* Rousi was not significantly related to geographic distance and altitude, which may be attributed to the genetic exchange within the sampled area. It is also possible that the high genetic diversity of Chinese sea buckthorn is due to its dioecious and wind-borne characteristics, the rich genetic variation of the species may expand its distribution range and facilitate adaptation to new environments (Nybom, 2004; Wang et al., 2014).

The rate of genetic variation among the 10 *H. rhamnoides* subsp. *sinensis* Rousi populations was 7%, and the rate of genetic variation within the populations was 93%, indicating that the genetic variation of Chinese sea buckthorn mainly occurred within the populations. It may be due to the stochastic nature of plant genetic diversity caused by geographic barriers and the diversity of the environments in which they are located, plants may undergo different types of variation patterns, such as continuous variation and random variation, in response to geographic changes (Liu et al., 2020). It is also found that *H. rhamnoides* subsp. *sinensis* Rousi from different geographic populations had a low degree of differentiation, and more genetic variation originated from different individuals within the populations (Wang et al., 2021a). The investigatedion of Chinese sea buckthorn at different altitudes by using ISSR molecular markers also indicate that *H. rhamnoides* subsp. *sinensis* Rousi at different altitudes had little spatial distance but showed obvious genetic diversity and genetic differentiation among populations (Chen et al., 2004). The genetic similarity of the populations in the 10 regions of Qinghai Province was relatively high. Thus, the appropriate inclusion of *H. rhamnoides* subsp. *sinensis* Rousi populations from other provenance should be considered in the future breeding of seedlings in Qinghai Province to enrich the genetic diversity.

## 5 Conclusion

In this study, 41,804 SSRs were identified from transcriptome data of *H. rhamnoides* subsp. *sinensis* Rousi, and the distribution and frequency of motifs were characterized and evaluated, fifteen SSR markers were developed for *H. rhamnoides* subsp. *sinensis* Rousi with abundant polymorphisms and showed a moderate level of genetic diversity. Finally, fifteen polymorphic SSRs were selected to investigate the genetic variation and structure of ten Chinese sea buckthorn populations in Qinghai province. The findings revealed a high level of genetic diversity in *H. rhamnoides* subsp. *sinensis* Rousi. AMOVA revealed that individual genetic variation is the main source of variation *of H. rhamnoides* subsp. sinensis *Rousi*. PCoA and Genetic structure analysis revealed two different genetic groups of natural *H. rhamnoides* subsp. *sinensis* Rousi in Qinghai Province. In addition, Our findings can provide a basis for the further investigation of *H. rhamnoides* subsp. *sinensis* Rousi by quantitative trait loci mapping, association analysis, and molecular-assisted breeding. Which can also further the exchange of Chinese sea buckthorn germplasms among different areas in China and the introduction of new Chinese sea buckthorn varieties from abroad.

## Data Availability

The original contributions presented in the study are publicly available. This data can be found here: https://www.ncbi.nlm.nih.gov/search/all/?term=PRJNA449450.
